# Getting operating theatre metrics right to underpin quality improvement: understanding limitations of NHS Model Hospital calculations

**DOI:** 10.1016/j.bja.2023.03.032

**Published:** 2023-05-09

**Authors:** Chen Zhang, Jaideep J. Pandit

**Affiliations:** 1 Nuffield Department of Anaesthetics, Oxford University Hospitals NHS Foundation Trust, Oxford, UK; 2 St John's College, Oxford, UK

**Keywords:** efficiency, modelling, NHS management, operating theatre, quality improvement, statistics

## Abstract

The Model Hospital is an NHS online resource summarising performance data for, amongst other things, operating theatres categorised by NHS Trust and specialty. As an official source of information, it might be assumed that metrics, such as ‘average late start time’, ‘average early finish time’, and ‘average late finish time’, are calculated in a way to reflect performance in these domains, but this is not the case. These values are, respectively, only for those lists that start late, finish early, and finish late, with the number of lists in each category unreported. The Model Hospital reports utilisation appropriately as ‘touch time’ (the time delivering anaesthesia and surgery) but prefers a ‘capped’ measure, in which any touch time occurring in late finishes is ignored. The Model Hospital aggregates utilisations across lists in a mathematically invalid way, which leads to the assumption that small aliquots of unused time on lists can be combined to create larger time blocks, in which to complete more operations. We present alternative, more intuitive, and mathematically conventional methods to derive performance metrics using the same data. The results have implications for hospitals developing their own dashboards and international organisations seeking to create national databases for operating theatre performance.

**Editor's key points**

• Operating theatre performance is assessed using metrics, such as utilisation, start time, and finish time. The Model Hospital presents such data for hospitals or specialties in the UK.
• The authors point out that the method used to calculate metrics appears to be mathematically incorrect. For example, ‘average late start time’ is presented only as the time for those lists starting late, and utilisations are combined in a way that yields no variance.
• The authors suggest that UK Model Hospital data should be avoided in research analysis or in developing international performance dashboards.

‘Quality improvement’ in perioperative medicine and anaesthesia is generally defined in the clinical terms of careful management of physiological (mainly cardiorespiratory), biochemical (e.g. blood glucose), and neurological (analgesia and depth of anaesthesia) variables to yield positive clinical outcomes, such as enhanced recovery.^1^ However, this work takes place within an organisational framework, including the scheduling of operating lists and ordering of patients. Regardless of clinical excellence, quality improvement can only occur if this environment is optimal and supportive.^2^ For example, poorly planned lists can result in patient cancellation, and overruns with late finishes can lead to staff exhaustion, stress, and suboptimal performance.^3^

Describing operating lists by key performance metrics reflects the effectiveness of the hospital environment in being able to deliver quality care. A pertinent question is if there is a consensus around what these metrics should be. Moreover, anaesthetists should be involved in developing these metrics as experts who work in operating theatre and as an integral part of the specialty's stated perioperative quality improvement programme. In this paper, we show how ‘subcontracting’ performance metric development to others has led to erroneous or misleading measures. If these are mistakenly used to plan services or rank hospitals, this would have the potential directly to impact adversely on the delivery of anaesthetic-surgical care.

In the UK, the superficial appearance of a consensus on key operating theatre metrics is provided by the Model Hospital of the Model Health System.^4^ This arose out of the review by Lord Carter of Coles, whose aim was to benchmark quality and productivity across NHS hospitals.^5^ NHS employees (who can access the data) and others can obtain current and historical summaries on how their service is performing by apparently relevant performance indicators. The operating theatres section within the Model Hospital is based on data provided regularly by individual Trusts in a specific format. The outputs include the metrics: utilisation, start and finish times, and intercase downtime. ‘Caterpillar’ plots (bar charts ordered from best-to worst-performing Trust in the given domain) permit comparison across organisations.

There is no dispute that in principle these metrics (utilisation, start, finish, and downtimes) are relevant, but it is their manner of calculation that raises concerns. For example, we have discovered that ‘average late start time’, as reported by the Model Hospital, does not mean the average start time across all lists but in fact the average only for those lists starting late. A previous NHS report into operating theatre productivity^6^ was found to contain important shortcomings in analyses and some basic errors in calculation.^7^ This justifies an independent scrutiny of how the Model Hospital operating theatre metrics are derived, especially as there are no peer-reviewed publications based on or emanating from the Model Hospital metrics. The findings have implications beyond the UK NHS: other countries seeking to develop national databases should not repeat these errors, and individual hospitals developing their own dashboards should not simply ‘cut and paste’ the Model Hospital's methods.

## Critical analysis of the Model Hospital operating theatres section

### Model Hospital dashboard view

The landing page of the Model Hospital website presents data for operating theatres' performance by Trust, or by specialty within a Trust, under key domains that include start and finish times and utilisation. Supplementary material 1 illustrates an example view, along with a hypothetical example dataset.

### Model Hospital's start and finish time metrics

If we were told that an employee ‘*on average arrived for work* 45 min *late*’ or ‘*on average clocked off work* 60 min *early*’, we might imagine that this was representative of their (poor) performance. We would be surprised if later we learned that these statements reflected only those rare occasions when the employee was late or has left early. Yet, this is exactly how the Model Hospital treats these timing metrics.

Moreover, it is well established that using means *vs* medians can greatly skew the results, as outlier values have disproportionate influence. The more mathematically conventional approach is to aggregate all the start and finish time data without selection bias and to report the median for the distribution of values (as better than mean for non-normal data), along with the variance (inter-quartile range and range). Supplementary material S1 shows how the same source data provide a sharply contrasting picture of performance when calculations are undertaken conventionally this way, as opposed to the Model Hospital methods.

These differences matter greatly because they create a potential for misunderstanding and disconnect between, on the one hand, national teams and regulators who currently rely on the Model Hospital as their reference and, on the other hand, local teams or Trusts.^8^ A Trust could almost eliminate late starts, yet find the Model Hospital's statistic unaffected, or even worsened, because only the late start time of the remaining single late running list will be reported.

The Model Hospital presentation of list finish times is similarly problematic but can confuse in one further respect. A specialty may be described, for example, as both ‘*finishing early on average by* 35 min’ and ‘*finishing late on average by* 45 min’. These would seem to be mutually exclusive things: if one finishes early, it is clearly impossible to be late, and *vice versa*. The explanation is that the Model Hospital is, respectively, reporting the average times for all the lists that team that finished early, and then the average times for all the lists that finished late. Because finishing exactly on time is impossible, all teams and hospitals will always be described as having some value of early finish (*x* minutes) and late finish (*y* minutes) for their specialties.

### Model Hospital's utilisation metrics

Utilisation is a fraction (percentage) that describes use of a resource. If a team is scheduled 10 h in operating theatre and its total hours of work 8 h (from when they started to when they finished is 8 h), their utilisation of time is 80% (8/10 h). This can be termed ‘raw’ or ‘crude’ utilisation because it does not take into account the gaps between cases when no anaesthesia or surgery is performed; operating theatres are cleaned, equipment prepared, or teams wait for patient arrival. This is the ‘intercase downtime’, and ‘touch-time utilisation’ subtracts the proportion of time spent in these gaps from the raw utilisation value. So, if this team's downtime was 2 h, then their touch-time utilisation is 60% (6 of 10 h spent in patient contact). These principles are well established and summarised in open resources^9^ and in standard texts on operating theatre management.^10^

Note that, in fact, there is more than one method of calculating intercase downtime. Two methods are shown in Supplementary material S2. The chosen method will influence the precise numerical value of touch-time utilisation.

The Model Hospital goes a step further in calculating the utilisation metric by trying to take into account *when* the team did the work. The underlying argument is that teams should ideally do the work within the specific time slots allotted to them (e.g. between 9 a.m. and 5 p.m.), and any work outside these time slots (i.e. overruns after 5 p.m.) should not be counted towards a utilisation metric. This is the principle of ‘capping’ utilisation in the Model Hospital's calculations.

Thus, touch-time utilisation will be lower than raw utilisation and capped utilisation will be lower than touch-time utilisation because some lists will invariably finish late. Supplementary material S3 shows in more detail as a graphic how the different types of utilisation (capped *vs* uncapped; raw *vs* adjusted for downtime) can yield different results for similarly performing teams.

Thus, the Model Hospital's preferred metric is decidedly conservative in its numerical value for utilisations. We see as follows how, along with the method of aggregating values across several lists, this weaves into its emphasis on ‘potential gains’ within the system. For purposes of interpreting team performance, there is no need to cap the utilisation metric because late finishes, if reported properly, will reflect that any high utilisation values are artificially high because of overruns.

### Aggregating the data across lists in the Model Hospital to estimate potential gains

Central to the Model Hospital approach is to estimate ‘potential gains’ as being the number of additional patients that hospitals could treat surgically, if they could improve their operating theatre utilisations. However, the method used to create this potential gain statistic is so mathematically invalid that it could not be used in a scientific paper.

Utilisation is a fraction, and the Model Hospital combines the fractions, as shown in Box 1. (For purposes of this discussion, whether this is capped or uncapped utilisation is not relevant; it is the method of aggregation that is the focus of the critique here.) This is incorrect. The Model Hospital is regarding the time allocated to a specialty across all its lists as single statistic (denominator), and then summing all the time used as the numerator. A graphic depiction of this method is shown in Supplementary material S4.

Box 1 **Model Hospital steps in calculating utilisation for a team or hospital where there are several lists (N1, N2, N3, etc.), each with an allocated time (in minutes, b, d, f, etc.), of which a certain time is actually utilised (a, c, e, etc.). The utilisation for a given list, (e.g. N1) is of course a/b, but the aim is to represent the utilisation of the team or hospital as a whole.**

Step 1

 Utilisation of list *N*1= $\frac{a}{b}$

 Utilisation of list *N*2= $\frac{c}{d}$

 Utilisation of list *N*3= $\frac{e}{f}$

 Etc.…

Step 2

 Aggregate utilisation of team or hospital= $\frac{a+c+e\ldots }{b+d+f\ldots }$.

Alt-text: Box 1

Moreover, this method provides no estimate of sample variance, but instead a single numerical value and hence no further statistics are possible. The method is akin, in a clinical trial, to estimating the efficacy of anti-hypertensive therapies by first summing the control blood pressures of all patients in the pre-treatment group (e.g. 40 000 mm Hg), and then comparing it with the post-treatment total (e.g. 30 000 mm Hg) and claiming a 25% efficacy (no standard deviations or measures of variance). No clinical trials are analysed in this way because it is invalid to do so. If the Model Hospital's method is unacceptable for other types of scientific data, then its use for equally important operating theatre performance data cannot be justified.

The mathematically conventional method of combining individual list utilisation values is shown in Box 2. In essence, the method converts the fractions to decimal notation and yields a median, interquartile range, and range for values (see Supplementary material S4 for a graphic depiction of this alternative method).

Box 2 **Conventional method of combining data that are fractions.**

Step 1

 Utilisation of list *N*1= $\frac{a}{b}$

 Utilisation of list *N*2= $\frac{c}{d}$

 Utilisation of list *N*3= $\frac{e}{f}$

 Etc.…

Step 2

 Convert *N*1 fraction to decimal notation (e.g. 0.83).

 Convert *N*2 fraction to decimal notation (e.g. 0.84).

 Convert *N*3 fraction to decimal notation (e.g. 0.81).

 Etc.…

Step 3

 Express the result as the median (inter-quartile range; range) of the value.

Alt-text: Box 2

**Fig 1:**
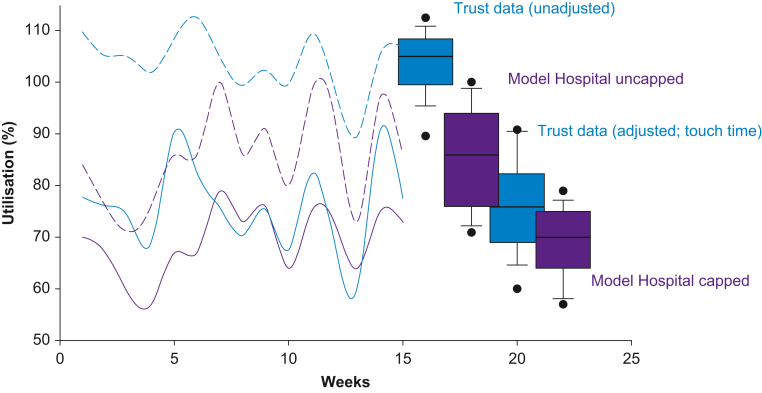
Comparison of the Model Hospital and alternative methods for calculating utilisation for real data for a single specialty in one Trust over 15 weeks. The Model Hospital results (purple; means) are shown for capped (solid line) and uncapped (dashed line) touch-time utilisation. The Trust results (blue; medians) are shown for raw unadjusted (dashed line) and adjusted for touch-time (solid line) utilisation. Note that there is no simple mathematical relationship between any of the four ways of calculating utilisation. In the box plots, the horizontal line is the median. The edges of the box are the 25th and 75th centiles, and the error bars are the 10th and 90th centiles.

Figure 1 shows the utilisation of a single specialty in a hospital, where the same source data are calculated using the Model Hospital and conventional methods. Differences arise because of the different methods of calculating utilisation (capping; Supplementary material S3) and intercase downtimes (Supplementary material S2), and of aggregating these (Supplementary material S4).

This unique approach to aggregating utilisation underpins the Model Hospital's estimation of ‘potential gains’: the ‘additional number of cases that there is capacity to treat’. The logic (which we will show is flawed) runs as follows. First, use the calculation of Box 1 to estimate the *unused* total capacity (i.e. the difference between the sum of the numerator and denominator values in Step 2; this may be 1200 min). Next, estimate the average duration of a case in the specialty (this may be 60 min). Finally, divide the first by the second to yield 20 extra cases as the potential gain that could/should have been done (see Supplementary material S5 for an illustration of this method). Higher potential gains act as a driver for hospitals to try to improve their performance. Potential gains will be higher; the lower the value for utilisation. Hence, capped utilisation yields a higher potential gains statistic.

The self-evident error in this approach is that ‘time’ is not a commodity that can be stored, saved, or combined into larger aliquots for future use. Small numbers of ‘spare’ minutes remaining on disparate lists cannot be added together to create many spare hours. If 5 min is wasted across each of 100 operating lists, it does not mean that we have 500 useful minutes of ‘spare capacity’ for surgery. Yet, this is exactly what the Model Hospital's method in Box 1 is designed to do.

## Discussion

This short review helps understand how the Model Hospital metrics are calculated, and in doing so it reveals their limitations and several mathematical errors. Because the measurement of performance directly influences clinical care, as it is used by regulators and others to plan services, the methods used should adhere to the same high standards as statistics for clinical research. Indeed, it is a concern that the Model Hospital metrics, emanating as they do from an authoritative national body, may be used as a ‘gold standard’ against which other research is judged. For example, a researcher using the process in Box 2 correctly to combine utilisations across lists may be required to revise their methods to the ‘official’ yet mathematically incorrect process of Box 1.

Whilst there are no direct international comparisons for the Model Hospital, there is clearly interest in developing these. The Canadian Institute for Health Information published an exploratory document,^11^ and in 2020 several German medical professional societies agreed a document relating to operating theatre procedure timings.^12^ The direction of travel is to create national databases to compare hospitals; hence, it is important that errors of calculation are not repeated.

It is notable that, to our knowledge, none of the methods used by the Model Hospital operating theatres has been subjected to peer review as journal publications. (Our current review is probably the first to do so.) Specifically, the notion of ‘capped utilisation’ is found nowhere in the now extensive literature on operating theatre management.^13^

There are reasons why capping may not withstand scrutiny. As discussed, it maximises the estimate of potential gains (additional number of patients who could have surgery). However, teams who overrun have already completed the extra cases. Capping disregards their overrun as contributing to utilisation and, in effect, conveys the message that more cases could be completed than have been. Clearly, this is wrong; these teams cannot realise the gain *because they have already completed the cases*. It is simply the utilisation that is being artificially capped, not the actual work or the patients.

A second limitation of capped utilisation is that it assumes that there are indeed fixed, immutable start and finish times to operating lists.^14^ On one level, teams clearly need to know when to start a list and when they can cease work; staff cannot just turn up and leave when they like. Also, regardless of the prevailing funding model, hospital income is predicated in part on there being certain hours allocated for elective surgery, and clear start and finish times help define this. In turn, staff contracts are time-sensitive (e.g. consultant contracts specify the hours of operating theatre time in ‘direct care activity’). Also, staff can claim overtime payments, triggered reference to the stated finish time.^15^

That all said, start and finish times are arbitrary, with no national standard. ‘Late’ starts and ‘early’ finishes only matter if they curtail surgical activity.^16^ For example, if a team starts an hour late and opts to finish an hour late to complete all the work, this has little or no adverse impact, and uncapped utilisation reflects this (Fig. 1). ‘Staggered start’ times have been described^17^ and modelled,^18^ which take into account the reality that there is uncertainty in key information, such as intensive care bed state, or equipment availability. Indeed, start time has probably evolved to being the time for WHO team huddle, only after which is the patient sent for.^19^ Similarly, finish times demonstrate variability (by normal, log normal, or Weibull distributions), requiring frequent adjustments to optimise list scheduled durations to optimise efficiency.^20^ With the introduction of ‘high-intensity’ (HIT) lists with overlapping surgery on parallel lists, the notion of a scheduled start time becomes even more redundant, as one of the parallel lists, by definition, always starts ‘late’ and the other list finishes ‘early’.^21^^,^^22^

Our technical commentary on the Model Hospital operating theatre metrics is not a criticism of its underlying intention, which was to highlight the importance of data in clinical operational decision-making and should not detract from the considerable service the resource has already provided. When conceived, it was an ambitious and unique project to summarise an entire country's operating theatre performance data. The deficiencies we identify may represent mathematically necessary shortcuts the Model Hospital had to take when dealing with the sheer volume of data, or may have arisen as a compromise because some Trusts submitted data in non-standard formats. Whether now to correct or improve the entire basis of the Model Hospital calculations is a separate question. A pragmatic compromise may be a two-step process of analysis. The first step is the ‘coarse filter’ of the Model Hospital that facilitates a consistent, if crude, national benchmark. The second step is the ‘fine filter’ of individual Trust dashboards, which use more conventional and accurate calculations and address local priorities with more granularity. Regardless, the proper understanding of operating theatre performance metrics is a prerequisite for quality improvement in perioperative care.

## Authors’ contributions

Study design: JJP.

Data collection: CZ.

Primary data analysis: JJP.

Checking of data analysis: CZ.

Drafting of paper: JJP.

Amendment of drafts of paper: CZ.

Finalisation of paper after discussion: both authors.

## Declaration of interest

The authors declare that they have no conflicts of interest.
