# Supplementary material for: Getting operating theatre metrics right to underpin quality improvement: understanding limitations of NHS Model Hospital calculations
Source: Br J Anaesth. 2023 May 9;131(1):130–4. doi: 10.1016/j.bja.2023.03.032 (PMC10308435; doi:10.1016/j.bja.2023.03.032)
Supplement: Multimedia component 1 [file mmc1.docx]

**Online Supplement S1: Model Hospital landing page metrics**

**Table S1**. Example table resembling the view on the landing page of Model Hospital for the key theatres data and (last column) results of an alternative approach to the metrics (by median (IQR [range]). Explanation in text. The intercase downtime is the gap between cases when no anaesthesia or surgery occurs; also called ‘gap time’ or ‘turnover time’. Utilisation is a percentage (fraction) of scheduled theatre time used, which is presented separately as ‘capped’ and ‘uncapped’ ‘touchtime’ utilisation. The original dataset from which values in each column can be derived are shown below the table (*n = 20 lists’ **n = 4 lists). Note that n of lists used to calculate metrics is not given by the Model Hospital view.

| Metric | Model Hospital view | Alternative method for same dataset |
| --- | --- | --- |
| average late starts (in minutes) | 28* | -4 (-6 to -3 [-9 to 82])* |
| average early finishes (in minutes) | 46* | n/a (encompassed in row below) |
| late finishes (termed ‘average unplanned session extension’) | 65* | 10 (-47 to 65 [-51 to 70])* |
| average intercase downtime (in minutes) | 82.5** | 14.3 (-9.6 to 24.3 [7.2 – 32])** |
| capped touchtime utilisation (%) | See text and Fig.1 (1^st^ column) | See text and Fig. 1 (adjusted utilisation, last column) |
| uncapped touchtime utilisation (%) | See text and Fig. 1 (2^nd^ column) | See text and Fig.1 (raw utilisation, last column) |
|  |  |  |

The data points used for ‘average late starts’ across 20 lists are: -6, -2, -5, -7, -4, -5, -9, -3, -6, -7, -3, -4, -4, -7, -4, -5, -2, 1, 1, 82. These yield an average of 38; and a median (IQR [range]) of -4 (-6 to -3 [-9 to 82]).

The data points used for ‘average early finishes’ and ‘average late finishes’ across 20 lists are: -40, -41, -42, -43, -44, -46, -47, -48, -49, -50, 60, 61, 62, 63, 64, 66, 67, 68, 69, 70. These yield an average early finish of 45 and an average late finish of 65 min; and median (IQR [range]) of 10 (-45 to 65 [-50 to 70]).

The data points used for ‘average unplanned session extension’ (average late finishes) across 4 lists are as follows. The 4 lists, A-D, have respectively, 3, 4, 5 and 6 cases per list. The downtimes between successive cases are (note that the last case has no downtime after it): List A 8,7; List B: 10,12,15; List C: 15,16,17,18; List D: 25,28,32,45,55. This yield median downtime values per case as: List A 7.5; List B 12; List C 16.5 and List D 32.
