# Supplementary material for: Getting operating theatre metrics right to underpin quality improvement: understanding limitations of NHS Model Hospital calculations
Source: Br J Anaesth. 2023 May 9;131(1):130–4. doi: 10.1016/j.bja.2023.03.032 (PMC10308435; doi:10.1016/j.bja.2023.03.032)
Supplement: Multimedia component 2 [file mmc2.docx]

**Supplement S2: Different ways of averaging: intercase downtime**

There is consensus that intercase downtime is relevant: within any scheduled time period it is desirable that clinical activity is maximised and gap time minimised. For *N* lists (*N*1, *N*2, *N*3, etc) each with a different *n* of cases there will be *n*-1 gaps, each with its downtime between successive cases (*g*1, *g*2, *g*3, etc).

Yet there are two ways of ‘averaging’, each with a different interpretation.

The Model Hospital sums the gaps and performs the sequence of calculations shown in Box S2.1, which is the *average downtime per list*. Model Hospital does not report the average downtime between cases, but this can be estimated from knowledge of the mean number of cases per list for the team.

**Box S2.1**. Model Hospital steps in calculating intercase downtime (see Online Supplement S1 for a numerical example).

Step 1

For list *N*1, sum the gap times: ∑ (*g*1 + *g*2 + *g*3 + *g*n) = *G*1

For list *N*2 sum the gap times: ∑ (*g*1 + *g*2 + *g*3 + *g*n) = *G*2

For list *N*3 sum the gap times: ∑ (*g*1 + *g*2 + *g*3 + *g*n) = *G*3

….etc

Step 2

Model Hospital reported downtime = $\frac{G1+G2+G3\ldots}{N of lists}$

The alternative method is to calculate the mean intercase downtime per list (*IG*1, *IG*2, etc), and then aggregate each of these across lists (Box S2.2). This yields a different numerical value to Box S2.1.

**Box S2.2**. Alternative method of calculating intercase downtime.

Step 1

For list *N*1: $\frac{\sum(g1+g2+g3\ldots)}{n of cases}$ = mean intercase gap for list N1 IG1

For list *N*2: $\frac{\sum(g1+g2+g3\ldots)}{n of cases}$ = mean intercase gap for list N2 IG2

For list *N*3: $\frac{\sum(g1+g2+g3\ldots)}{n of cases}$ = mean intercase gap for list N2 IG3

….etc

Step 2

Aggregate the mean intercase downtimes = $\frac{IG1+IG2+IG3\ldots}{N of lists}$
