# Supplementary material for: Getting operating theatre metrics right to underpin quality improvement: understanding limitations of NHS Model Hospital calculations
Source: Br J Anaesth. 2023 May 9;131(1):130–4. doi: 10.1016/j.bja.2023.03.032 (PMC10308435; doi:10.1016/j.bja.2023.03.032)
Supplement: Multimedia component 3 [file mmc3.docx]

**Online Supplement S3: Explaining Model Hospital capped utilisation**

**Figure S3.1.** Explaining. Four hypothetical lists A-D with the same scheduled start (09.00) and finish (17.00) times (red lines; scheduled list time 8 h, 480 min), but which actually start and finish at different times. In each list, each gap between the cases (represented as grey bars) is 15 min (total gap time 45 min). List A starts and ends 55 min late. List B is identical except it starts and ends 55 early. List C is identical except it starts and finishes on time. List D starts, like A, 55 min late but finishes on time, with less total touchtime. The values for Model Hospital (capped and uncapped) metrics reflecting the respective performances are shown. In the last column are shown the values using the alternative method for utilisation, raw, unadjusted and in brackets, adjusted for intercase turnover time.


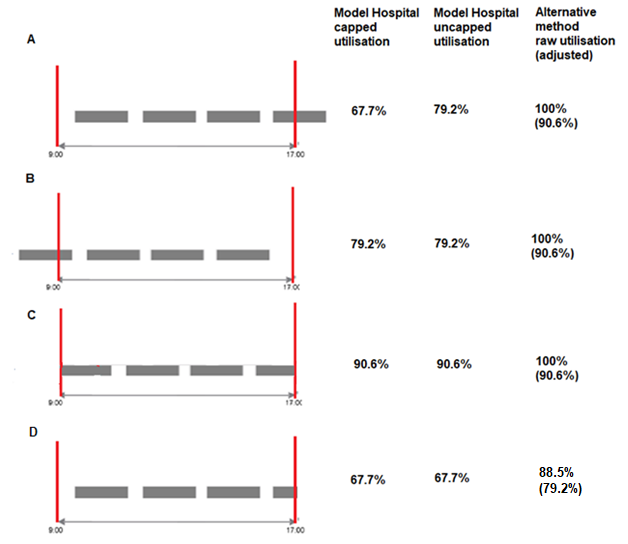


*Discussion*

Figure S3.1A shows a hypothetical list starting and finishing 55 min late. The denominator for the utilisation calculation is scheduled time of 8 h (480 min). In the Model Hospital’s ‘capped’ metric, touchtime in over-runs is not counted, hence yielding a value of 67.7%. Uncapped utilisation, which includes the touchtime in over-runs is higher, at 79.2%.

However, a limitation of the capped utilisation method is illustrated by Figure S3.1B. This list is identical in every respect to that in Figure 1A, except it starts and finishes 55 min early. Since Model Hospital’s method includes touchtime before the scheduled start time, but not after, the resulting capped utilisation of Figure S3.2B is much higher: 79.2%. In this example, the uncapped values of Figure S3.1A and Figure S3.1B are the same. The concern here is that for two lists which differ only in being ‘time-shifted’ with respect to scheduled start and finish times (n of operations, intercase downtimes, etc, all being the same), capped utilisation favours the one starting early over the one starting late; i.e., capped utilisation is sensitive to scheduled start and finish times but not to other measures of team performance or productivity.

This concern is underlined by now considering another identical list in Figure 3.1C, which starts and finishes to its scheduled time, but delivers the same quantum of work - and now has a very impressive capped utilisation 91%. Why three lists with the same fundamental performance and service delivery should be assigned such wide variation in a utilisation metrics ranging from 67.7% to 91% seems unjustified. Another limitation of using capped utilisation is shown by the team in Figure S3.1D, which curtails its work to avoid over-run and so delivers less total surgical/anaesthesia hours. By Model Hospital metrics, its performance in capped utilisation is identical to the team in Figure S3.1A, that works longer hours. It seems a flaw to assign the same utilisation performance to a team that works fewer-than-contracted hours, as to a team that works all its hours.

An alternative measure of utilisation would be insensitive to scheduled start and finish times, and instead reflect actual work delivered (last column of Figure S3.1). The values here represent the raw (unadjusted) utilisation as the numerator: the time period from the start of anaesthesia in the first patient to the completion (arrival in recovery) of the last patient. This is divided by the scheduled time (denominator, in minutes). The ‘raw’ value includes (i.e., ignores) the intercase downtimes, and can be adjusted to derive the touchtime by subtracting the respective intercase downtimes. The raw utilisation values in Figures S3.1A-C of > 90% do not imply that these are near-perfect lists; this simply reflects the reality that these teams have worked the scheduled hours. Other statistics, such as median start times or median early or late finishes (e.g., last column Table S1, Online Supplement S1) will reflect the ways in which these teams can improve performance.
