# Supplementary material for: Getting operating theatre metrics right to underpin quality improvement: understanding limitations of NHS Model Hospital calculations
Source: Br J Anaesth. 2023 May 9;131(1):130–4. doi: 10.1016/j.bja.2023.03.032 (PMC10308435; doi:10.1016/j.bja.2023.03.032)

**Online Supplement S4: Model Hospital vs conventional methods of aggregating utilisations**

**Figure S4.1**. Explaining how Model Hospital aggregates utilisations across lists for a specialty (or a hospital). The small buckets represent individual lists of different scheduled capacity (bucket size), and are filled (utilised) in different proportions. Model Hospital sums the individual capacities to create a total specialty capacity (the large, super-bucket), to yield the denominator. Then, the individual minutes of utilised time from each list are summed to yield the numerator. This is akin to pouring water from individual buckets into the super-bucket and assessing its degree of filling (here, 33% from the values given).


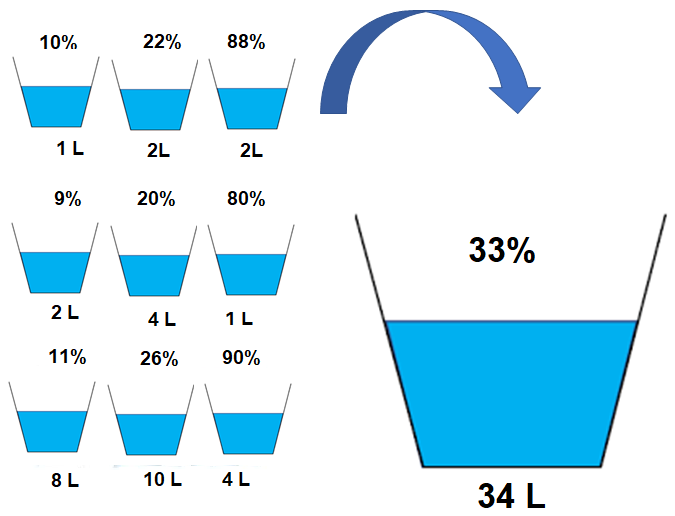


**Figure S4.2**. Alternative method of aggregating individual list utilisations. The same values as in Figure S4.1 are used for the smaller buckets. The median (IQR [range]) of all the individual utilisations is 22 (11- 82 [9 – 90])% (n = 9). The boxplot is shown in the right, with the horizontal red line representing an aspirational utilisation goal of 80%.


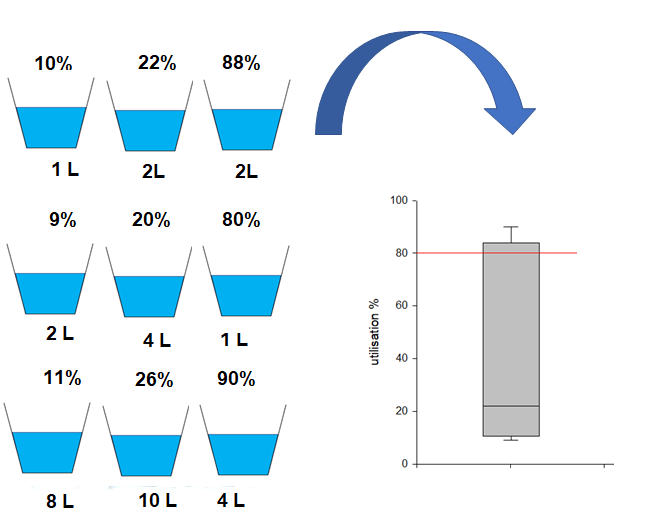

Supplement: Multimedia component 4 [file mmc4.docx]
