# Supplementary material for: Getting operating theatre metrics right to underpin quality improvement: understanding limitations of NHS Model Hospital calculations
Source: Br J Anaesth. 2023 May 9;131(1):130–4. doi: 10.1016/j.bja.2023.03.032 (PMC10308435; doi:10.1016/j.bja.2023.03.032)

**Online Supplement S5: Explaining Model Hospital’s method of estimating ‘potential gains’**

**Figure S5.1.** The four lists A-D are of 240 min scheduled duration (scheduled start to scheduled finish times; red lines). Each completes four cases of duration 50 min. There is a late start of 10 min and early finish of 15 min, and intercase downtime of 5 min per case. These gaps total 160 min across the lists and since the average case duration is 50 min, Model Hospital argues this represents a potential gain of 3 cases which could have been done, if all the time had been saved. The flaw in the logic is that in fact, even if all the gaps on any single list were eliminated (40 min per list), there is not time to accommodate even one extra case on any list. The individual, small ‘spare’ aliquots of time cannot be lumped together to create more time.


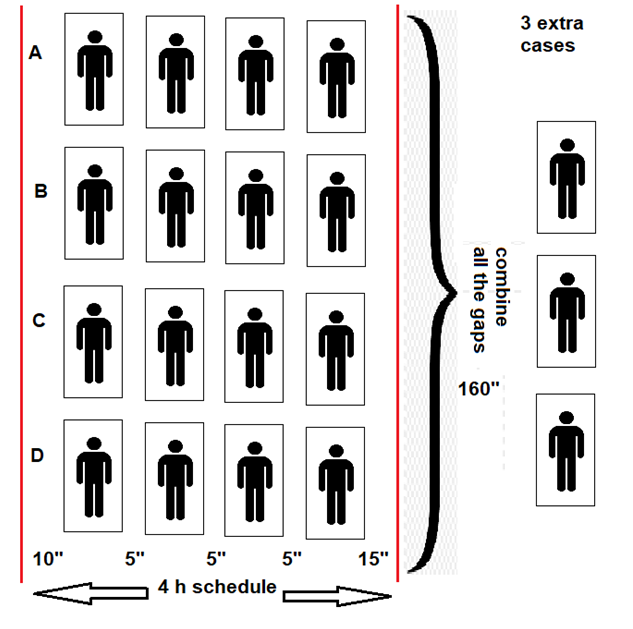

Supplement: Multimedia component 5 [file mmc5.docx]
